# Whole-Genome Sequence of Salmonella enterica Serovar Bispebjerg from Turkey Reveals Its Pathogenic Potential

**DOI:** 10.1128/mra.00043-23

**Published:** 2023-04-06

**Authors:** Alessia Tiengo, Massimiliano Orsini, Sara Petrin, Carmen Losasso, Alessandra Longo, Giulia Cento, Laura Ciot, Raffaella Ceruti, Veronica Cibin, Lisa Barco

**Affiliations:** a Italian National Reference Laboratory for Salmonellosis, Istituto Zooprofilattico Sperimentale delle Venezie, Legnaro, Padua, Italy; b Gesco SCA Laboratory, Cazzago San Martino, Brescia, Italy; c Epidemiology Department, Istituto Zooprofilattico Sperimentale delle Venezie, Legnaro, Padua, Italy; University of Maryland School of Medicine

## Abstract

We report the genome sequence of a Salmonella enterica subsp. *enterica* serovar Bispebjerg strain that was isolated from a turkey flock in 2011. The genome analysis of the strain, a rare and multihost serovar, revealed its pathogenic potential due to antimicrobial resistance and a plethora of Salmonella pathogenicity islands and virulence factors.

## ANNOUNCEMENT

In 2011, feces from a breeder turkey flock in Grosseto, Italy, were positive for Salmonella enterica subsp. *enterica* serovar Bispebjerg (1, 2). Although *S.* Bispebjerg is a multihost serovar (3), it has been rarely isolated, and the Italian Reference Laboratory identified only 2 strains in 2011 to 2020. The turkey isolate was maintained at −80°C in a cryobank until sequencing and then was cultured overnight in tryptic soy broth (Sigma-Aldrich) at 37°C, and genomic DNA was extracted with a DNeasy kit (Qiagen). One nanogram was used for next-generation sequencing (NGS) library preparation using the Illumina Nextera XT DNA library preparation kit (New England Biolabs, Ipswich, MA, USA). Sequencing was performed on the Illumina MiSeq platform, yielding 2,194,751 read pairs (2 × 250 bp reads; theoretical coverage, 219×). An additional 1.5 ng was used for the preparation of Nanopore libraries using a SQK-RBK004 rapid sequencing kit and sequencing using a FLO-MIN106 (R9) flow cell on a MinION device (Oxford Nanopore Technologies, UK); this yielded 21,268 raw reads (total size, 115.9 Mb; *N*_50_, 8,184 bp; theoretical coverage, 23×). Read contamination was checked with ConFindr v0.7.4 (4). Read cleaning by fastp v0.20.1 (5) and Nanofilt v2.8.0 (6) for Illumina and Nanopore data, respectively, returned 2,118,530 paired-end reads and 15,680 long reads, respectively, which were assembled by Unicycler v2.1.1 (7). Default parameters were used for all software tools unless otherwise noted. The final assembly consisted of two circular sequences of 5,023,030 bp (GC content, 52.2%) and 138,198 bp (GC content, 51.6%). The larger sequence was rotated using DFAST v1.2.18 (https://dfast.ddbj.nig.ac.jp), which also performed quality controls implementing CheckM v1.2.2 (8). This assessment indicated completeness of 99.52%, and the taxonomy check with FastANI v1.33 (9) indicated Salmonella enterica LT2 (GenBank assembly accession number GCA_001558355.2) as the closest match (average nucleotide identity [ANI], 98.846%). The larger sequence was annotated by PGAP v4.3 (10), which identified 4,720 protein-coding genes, 22 rRNAs, 84 tRNAs, and 2 CRISPR arrays for the longest sequence. Genome characterization with ResFinder and PointFinder v4.1 (11, 12) highlighted the presence of the *aac(6′)* gene and the *parC* T57S mutation, conferring resistance to aminoglycosides and nalidixic acid-ciprofloxacin, respectively, while SPIFinder v2.0 (13) identified at least 9 regions ascribable to Salmonella pathogenicity islands (SPIs) (SPI-1 to SPI-5, SPI-9, and SPI-12 to SPI-14). Finally, 106 virulence factors were identified using Abricate v1.0.1 (https://github.com/tseemann/abricate) and the Virulence Factor Database (VFDB) (14), with matches showing at least 80% coverage and identity; most factors were involved in adherence (*n* = 20) and the effector delivery system (*n* = 76). The smaller sequence, potentially containing 152 genes as detected by DFAST v1.2.18, was searched for origins of replication by PlasmidFinder v2.1 (15) and queried against the DoriC v10.0 database (16), which did not identify any known incompatibility group or plasmid origin. The absence of a replication origin sequence led us to suspect some assembly artifacts and to not further consider the sequence for submission.

### Data availability.

The genome assembly (GenBank accession number CP115551) and raw reads (SRA accession numbers SRR22904678 and SRR22904677) are available under BioProject accession number PRJNA916372. Preliminary assembly of the putative plasmid is available on request to the authors.
